# Effect of aromatic stimulation on CPAP adherence and sleep quality in patients with obstructive sleep apnea: a pilot study

**DOI:** 10.1186/s12906-025-05243-9

**Published:** 2026-01-09

**Authors:** Erina Ishimizu, Ayako Inoshita, Yo Suzuki, Masahiro Nakamura, Takatoshi Kasai, Fumihiko Matsumoto

**Affiliations:** 1 Department of Otorhinolaryngology, Juntendo University Graduate School of Medicine, 2-1-1 Hongo, Tokyo, Bunkyo - Ku 113-8421 Japan; 2 Sleep and Sleep - Disordered Breathing Center, Juntendo University Hospital, Tokyo, Japan; 3 Cardiovascular Respiratory Sleep Medicine, Juntendo University Graduate School of Medicine, Tokyo, Japan; 4 Department of Cardiovascular Biology and Medicine, Juntendo University Graduate School of Medicine, Tokyo, Japan

**Keywords:** Obstructive sleep apnea (OSA), Continuous positive airway pressure (CPAP), CPAP intolerance, Aroma oil, Sleep quality

## Abstract

**Purpose:**

Continuous positive airway pressure (CPAP) therapy remains the cornerstone of obstructive sleep apnea (OSA) treatment, considerably reducing the risk of cardiovascular complications and improving patient outcomes. However, adherence to CPAP therapy is a major challenge and poor compliance limits its efficacy. This study investigated the potential of aromatherapy, a noninvasive, cost-effective intervention, to improve CPAP adherence and enhance sleep quality in patients with OSA.

**Methods:**

A prospective observational pilot study was conducted in patients with obstructive sleep apnea who demonstrated poor CPAP adherence (< 70% usage and < 4 h/night). Participants were exposed to lavender or cypress aroma oil during sleep. Pre- and post-intervention subjective sleep measures Pittsburgh Sleep Quality Index (PSQI) and Epworth Sleepiness Scale (ESS), and objective CPAP usage metrics were collected. Normally distributed variables were analyzed using paired t-tests, and non-normally distributed variables were analyzed using the Wilcoxon signed-rank test. Statistical significance was set at *p* < 0.05. A post hoc power analysis was performed based on observed effect sizes for the primary outcomes.

**Results:**

Eight patients with OSA (mean age 53.5 years; 4 males, 4 females) participated in the study. Following 67 days of treatment, the median PSQI score significantly improved from 9.0 to 6.5 (*p* = 0.006), and the median ESS score decreased from 9.0 to 6.5 (*p* = 0.034). Additionally, CPAP for more than 4 h increased from 5.0% to 25.7% (*p* = 0.028). The median usage duration improved from 149 to 231 min (*p* = 0.018). No significant change in apnea hypopnea index (AHI) was observed during CPAP use, decreasing from 3.2 events/h to 1.3 events/h (*p* = 0.226), which is consistent with the understanding that appropriately applied CPAP maintains effective control of respiratory events.

**Conclusion:**

Aromatic stimulation with essential oils shows promise in improving both CPAP adherence and sleep quality, offering a novel approach to enhance OSA treatment efficacy.

## Introduction

Obstructive sleep apnea (OSA) is a common condition associated with significant morbidity and mortality, including excessive daytime sleepiness, automobile accidents, and cardiovascular complications such as hypertension and arrhythmias [1–3]. Continuous positive airway pressure (CPAP) therapy improves psychological and physical impairments in patients with OSA with sleepiness, fatigue, neurocognitive impairment, and depression. Moreover, it can prevent hypertension, adverse cardiovascular events, and arrythmia [1, 4]. CPAP is gold standard treatment for patients with moderate to severe OSA [5, 6].

However, the effectiveness of CPAP therapy is strongly dependent on treatment adherence. Optimal therapeutic benefit requires at least 4 h of nightly use on > 70% of nights, yet adherence rates remain suboptimal, ranging from 30 to 60% worldwide [7, 8]. Poor CPAP adherence is a major clinical challenge, leading to persistent symptoms, increased healthcare utilization, and higher risk of cardiovascular events. Common barriers include mask discomfort, nasal symptoms, dryness, pressure intolerance, and psychological resistance to device use [9]. Although interventions such as mask fitting optimization, humidification, nasal therapy, and behavioral support are routinely implemented, a considerable proportion of patients continue to struggle with CPAP acceptance and adherence [10]. Thus, alternative or adjunct strategies to improve tolerance and long-term adherence are urgently needed.

Alternative or adjunctive treatments for OSA include oral appliances, uvulopalatopharyngoplasty (UPPP) and upper airway surgeries [11], and hypoglossal nerve stimulation (HGNS) has emerged as an option for CPAP-intolerant patients [12]. Nevertheless, surgical approaches are invasive and limited to selected candidates [13], highlighting the need for non-invasive, patient-friendly methods to support CPAP use.

Recently, medical aromatherapy has been used to examine the impact of essential oil scents, such as lavender, cypress, and rose, on psychological and physiological states, garnering particular interest in their potential to enhance sleep quality [14, 15]. In clinical practice, aromatherapy has been introduced in various clinical settings, including palliative care, intensive care units, and bathing methods [16, 17]. However, despite these findings, little is known about whether aromatherapy can specifically support CPAP adaptation and adherence in patients with OSA, and no standardized approach currently exists for integrating aromatherapy into CPAP use. Given these considerations, this pilot study aimed to investigate whether aromatic stimulation could improve CPAP tolerability, enhance adherence, and improve subjective sleep quality in patients with OSA who exhibit difficulty with CPAP therapy.

## Materials and methods

### Patients

This prospective observational pilot study included eight CPAP-intolerant patients with OSA who had received CPAP treatment at the Sleep-Disordered Breathing Center of Juntendo University Hospital between March 8, 2019 and February 10, 2020. Patients were intentionally selected using a purposive sampling method according to the following inclusion criteria: a confirmed diagnosis of OSA; CPAP use for at least three months with an average nightly usage of less than 4 h; no nasal or mask-related problems; and absence of other sleep disorders.

Nasal endoscopy was performed in all patients to confirm the absence of nasal polyps, hypertrophic rhinitis, and olfactory fissure occlusion. The presence of olfactory disturbances was confirmed through interviews. The CPAP equipment used was Sleep mate®10 (ResMed, San Diego, USA) or Dream Station® (Philips Respironics, Amsterdam, NLD). The exclusion criteria were as follows: (1) age < 20 years, (2) nasal diseases with olfactory dysfunction, (3) a history of allergy to lavender or cypress oil, (4) no smell of aroma oil, (5) pregnancy, and (6) neuromuscular disease. This study was approved by the Ethics Committee of the Juntendo University Faculty of Medicine (E18-213) and conducted in accordance with the Declaration of Helsinki and Good Clinical Practice guidelines. Written informed consent was obtained from all participants.

### Aromatherapy

**Fig. 1:**
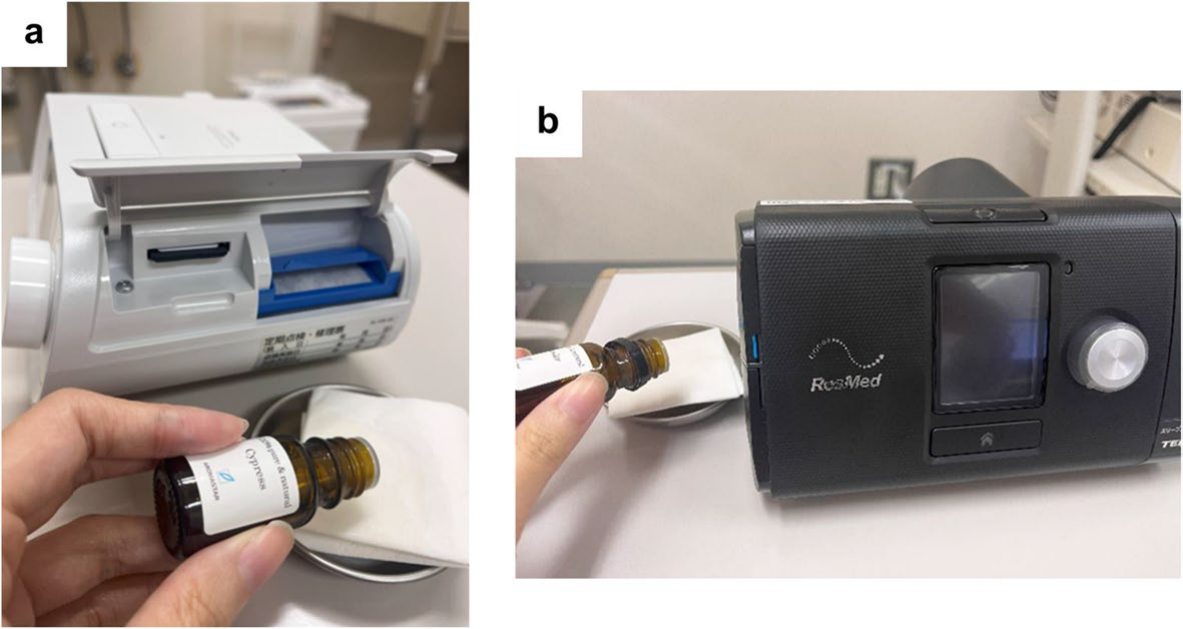
The actual setting of aroma oil. Aroma oils on a tissue of cotton were placed nearby CPAP filter. **A** Sleep mate®10, **B** Dream Station®

The patients were provided with specific instructions to place a tissue or cotton ball infused with one–three drops of aroma oil adjacent to their CPAP apparatus before commencing their nightly sleep regimen. The quantity of aroma oil applied was tailored to individual smell intensity preferences. Two distinct types of essential oil, lavender and cypress (100% pure&nature lavender® and 100% pure&nature cypress® AROMASTAR, Nagoya, JPN) were applied based on previous reports on their effects on good sleep [16, 18]. The methodology for administering these aroma oils was comprehensively elucidated during outpatient consultations with practical demonstrations utilizing the actual aroma oil (Fig. 1). Patients confirmed aroma use on CPAP nights via a daily diary, which was used to monitor compliance.

### Outcomes

For evaluation purposes, two established tools, the Epworth Sleepiness Scale (ESS) and the Pittsburgh Sleep Quality Index (PSQI), were administered both before and after the aroma oil trial [16–19]. Excessive daytime sleepiness was measured using the ESS, with scores of 11 or higher indicating excessive daytime sleepiness. To measure subjective sleep quality, a widely used self-administered questionnaire, the PSQI, was completed in the previous month. To assess the effect on sleep onset, we compared the sleep onset latency before and after using the oil. Sleep-onset latency was measured by self-reporting using the PSQI. Post-intervention assessments of ESS and PSQI were obtained approximately two months after initiation of the aromatherapy intervention.

The downloaded CPAP data were compared before the use of the aroma oil and the last two months of the trial. The following CPAP parameters were evaluated: (1) percentage of days with CPAP usage (%), (2) percentage of days with CPAP usage > 4 h (%), (3) average usage per night (min), and (4) average apnea–hypopnea index (AHI) on CPAP (events/h). CPAP usage metrics were likewise evaluated over the final two months of the aromatherapy period. No additional interventions, such as therapeutic education, phone calls, proactive follow-up, or telemonitoring, were provided.

### Statistical analyses

No statistical sample size calculation was conducted, as this was an exploratory pilot study.

A post hoc power analysis was performed using the observed effect sizes for the main outcomes (PSQI, ESS, and CPAP adherence). However, post hoc power analyses indicated that the study had 91%, 66%, and 98% power to detect the observed differences in PSQI, and ESS,　and CPAP usage (> 4 h), respectively.

Each score in the survey items was compared using the *t*-test for normally distributed continuous variables and the Wilcoxon signed-rank test for non-normally distributed continuous variables. A value of *p* < 0.05 was considered to be statistically significant. All values are shown as the median interquartile range.

## Results

Among 388 patients undergoing CPAP therapy, 8 met the inclusion criteria and were enrolled in this study. The median age was 53.5 years [50.3–71], and four of the eight were male (Table 1). The median duration of CPAP use with aroma usage was 67 days [46–116]. Patients applied the aroma oil on the nights they used CPAP and documented its use in a daily diary for compliance monitoring. The results are summarized in Table 2.

**Table 1:** Patient characteristics

Variable	Value
Age, years	58.4 ± 11.3, 53.5 [50.3–71]
Sex	
Male, n (%)	4 (50.0)
Female, n (%)	4 (50.0)
Complications	
Hypertension, n (%)	3 (37.5)
Diabetes, n (%)	1 (12.5)
Heart failure, n (%)	2 (25.0)
Respiratory disease, n (%)	0 (0.0)
AHI at OSA diagnosis (events/hour)	43.1 ± 13.2, 46.8 [33.4–50.5]
Duration of CPAP use (month)	26.6 ± 32.4, 10 [8.5–34]
PSQI score	8.8 ± 2.5, 9 [7.5–9.5]
ESS score	9.4 ± 5.0, 9 [5.8–12]

Data are presented as mean ± SD, median [IQR], or n (%)

*AHI* apnea hypopnea index, *OSA* obstructive sleep apnea, *CPAP* continuous positive airway pressure

**Table 2:** PSQI,ESS, and CPAP use before and after using aroma oil

Variable	Baseline	Post-treatment	*p*-value
PSQI score	9.0 [7.5–9.5]	6.5 [4.8–7.3]	0.006 *
ESS score	9.0 [5.8–12]	6.5 [4.3–11]	0.034 *
Sleep onset latency (minutes)	15 [9–23]	9 [5–10]	0.390
Download date from CPAP			
Percent days with CPAP usage (%)	43.6[26.9–68.3]	66.1 [63.2–77.8]	0.043 *
Percent of days with CPAP usage > 4 h (%)	5.0 [1.4–8.5]	25.7 [14.7–32.2]	0.028 *
Average usage (min)	149 [105–191]	231 [184–276]	0.018 *
Average AHI on CPAP (events/h)	3.2 [1.8–4.2]	1.3 [1.0–3.0]	0.226

Data are presented as median [IQR]

*PSQI* Pittsburgh Sleep Quality, *ESS* Epworth Sleepiness Scale, *CPAP* continuous positive airway pressure, *AHI* apnea hypopnea index

**Fig. 2:**
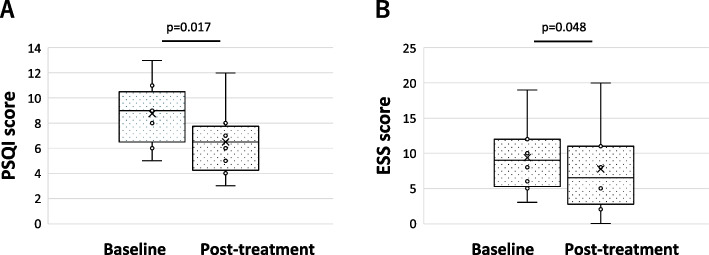
Changes before and after treatment with aroma oils in questionnaires. **A** PSQI score showed significant improvement after the aroma therapy from 9.0 [7.5–9.5] to 6.5 [4.8–7.3 (*p* = 0.006). **B** ESS score showed significant improvement after the aroma therapy from 9.0[5.8–12] to 6.5[4.3–11 (*p* = 0.034)

The PSQI for sleep quality significantly decreased from 9 [7.5–9.5] to 6.5[4.8–7.3] (*p* = 0.006) (Fig. 2A). The ESS, which was for subjective symptoms of daytime drowsiness significantly decreased from 9 [5.8–12] to 6.5 [4.3–11] after oil use (*p* = 0.034) (Fig. 2B).

**Fig. 3:**
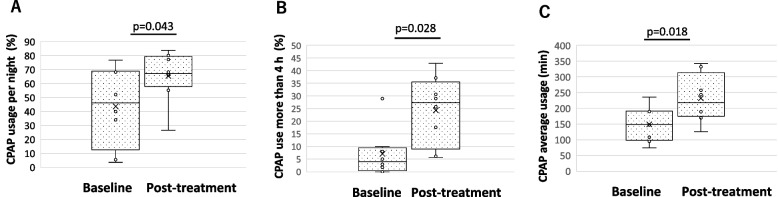
Changes before and after treatment with aroma oils in CPAP data. **A** CPAP usage per night showed significant improvement after the aroma therapy from 43.6 [26.9–68.3] % to 66.1 [63.2–77.8] % (*p* = 0.043). **B** CPAP usage per night more than 4 h showed significant improvement after the aroma therapy from 5.0 [1.4–8.5] % to 25.7 [14.7–32.2] % (*p* = 0.028). **C** CPAP average usage time showed significant improvement after the aroma therapy from 149 [105–191] minutes to 231 [184–276] minutes (*p* = 0.018)

As for CPAP, the usage rate increased from 43.6% [26.9–68.3] to 66.1% [63.2–77.8] (*p* = 0.043) (Fig. 3A), the percentage of days used for more than 4 h from 5.0% [1.4–8.5] to 25.7% [14.7–32.2] (*p* = 0.028) (Fig. 3B), and the average duration of use from 149 min [105–201] to 231 min [184–276] (*p* = 0.018) (Fig. 3C), showing significant improvement in all parameters. No significant differences were observed in AHI under CPAP use from 3.2 events/h [1.8–4.0] to 1.3 events/h [1.0–3.0] (*p* = 0.226) and sleep onset latency from 15 min [9–23] to 9 min [5–10] (*p* = 0.390). None of the patients had unpleasant feelings with the smell of aroma oil or nasal symptoms such as nasal obstruction.

## Discussion

In this pilot study, we investigated whether aromatic stimulation could improve the tolerability of CPAP in patients with OSA. Several notable findings emerged. First, both ESS and PSQI scores improved after exposure to aroma oils, suggesting enhanced subjective sleep quality. Additionally, total CPAP usage, the proportion of nights with > 4 h of use, and average CPAP usage duration all increased. These results indicate that aromatic stimulation may have a positive influence on CPAP adherence. Importantly, improvements in subjective sleep quality and increased CPAP usage were observed in parallel, suggesting a potential bidirectional interaction whereby more restorative sleep may facilitate mask tolerance, while increased CPAP use may further enhance sleep quality.

Although CPAP is the first-line therapy for moderate to severe OSA, mask intolerance can limit its effectiveness [20]. Alternative therapies such as OA confer certain cardiovascular benefits but may cause side effects such as jaw discomfort [21–23]. Surgical options, including uvulopalatopharyngoplasty (UPPP) and HGNS, may improve AHI but are only suitable only for selected patients and carry risks of complications [24–28]. Therefore, developing effective noninvasive strategies to enhance CPAP adherence remains clinically important. Importantly, improvements in subjective sleep quality and increased CPAP usage were observed in parallel, suggesting a potential bidirectional interaction whereby more restorative sleep may facilitate mask tolerance, while increased CPAP use may further enhance sleep quality.

This study indicated that the effects of aromatic stimulation may have positively influenced sleep centers. The olfactory system plays a pivotal role in the mediation of the effects of aromatherapy. Odor molecules are transmitted from the olfactory epithelial mucosa in the nasal passages to the olfactory bulb, where they initiate a cascade of signals that traverse various brain regions, including the anterior piriform cortex, anterior olfactory nucleus, amygdala, hypothalamus, and entorhinal cortex. From the piriform cortex, these signals are relayed to the frontal olfactory cortex of the cerebral cortex, where they are cognitively processed as distinct odors. Notably, the amygdala and hypothalamus govern emotions, whereas the hippocampus is associated with memory [29]. In addition, because the hypothalamus contains sleep and autonomic nervous centers, odor stimuli may affect sleep quality and autonomic nervous system activity [29]. The results of this study suggest that the transmission of lavender and cypress oil, which are considered to have good odors, through these pathways stimulates sleep and autonomic nerve centers, resulting in deep sleep, which may be involved in improving CPAP adherence and sleep quality. Odor stimulation may also increase the parasympathetic nervous system activity, which may reduce the physical and emotional stress caused by CPAP use. Okabe et al. examined healthy subjects immediately after rose odor stimulation during REM sleep and asked them about their dreams; many responded that they had bad dreams [14]. However, in our results, although dreams were not included in the survey items and olfactory elements were not the same components, the PSQI results indicated that olfactory stimulation improved sleep quality. Furthermore, pleasant odors induce a deep "sniffing response" during sleep, and even the anticipation of being presented with pleasant odors can induce a sniffing response [30]. The present study demonstrated an improvement in CPAP adherence, suggesting that the aroma oil load during sleep, which is perceived as pleasant, may have facilitated and stabilized breathing.

The results of this study suggest a new perspective that low-cost olfactory stimulation is potentially effective for improving sleep quality in CPAP-intolerant patients.

Previous studies have reported a high prevalence of olfactory dysfunction in patients with OSA, correlating with disease severity [31, 32], potentially due to nasal obstruction, impaired mucociliary clearance, or neuronal injury from intermittent hypoxia [33]. CPAP therapy may improve olfactory function by restoring nasal airflow, reducing inflammation, and mitigating hypoxia-related neuronal damage [31, 32]. Although our participants did not report olfactory dysfunction, these findings support the rationale that olfactory stimulation may complement CPAP therapy by engaging neural pathways involved in sleep regulation and adherence.

Pearl et al. demonstrated that respiration-triggered olfactory stimulation could reduce OSA severity, as measured by AHI and oxygen desaturation indices [33]. In contrast, our study applied continuous exposure to pleasant aroma oils during sleep. Despite differences in stimulation method, both studies support the notion that olfactory input can positively influence sleep and respiratory parameters. In our study, improvements in PSQI and ESS scores were accompanied by increases in total CPAP usage, proportion of nights with > 4 h of use, and average nightly usage duration, suggesting that continuous aromatic stimulation may enhance sleep quality and facilitate adherence to CPAP therapy. Previous studies examined olfactory function in patients with OSA, showing a higher prevalence of olfactory dysfunction compared to healthy controls and correlations with AHI [34, 35]. Causes may include nasal obstruction, impaired mucociliary clearance from chronic upper airway inflammation, or neuronal damage due to intermittent hypoxia [36]. The results of this study suggest a new perspective that low-cost olfactory stimulation is potentially effective for improving sleep quality in CPAP-intolerant patients.

This study has several limitations. Sleep electroencephalography recordings were not incorporated into our study protocol, thereby precluding the opportunity for an objective assessment of sleep patterns and characteristics. The sample size was small, and no a priori sample size calculations were performed, and no control group was included. Baseline olfactory function was not formally tested, and the amount of aroma oil used was not standardized. Nevertheless, we cannot exclude the possibility that the use of aroma oils itself enhanced patients’ motivation or awareness toward CPAP therapy, indirectly contributing to better adherence. As a pilot study, further research is needed, but our findings suggest that low-cost olfactory stimulation may represent a promising adjunctive approach to enhance sleep quality and CPAP adherence in patients with poor compliance.

## Conclusion

Aroma oil appears to have the potential to enhance sleep quality, improve daytime sleepiness, and promote greater adherence to CPAP therapy in patients with OSA who experience difficulty with CPAP treatment. A plausible mechanism underlying these effects is the impact of olfactory stimulation on sleep and autonomic regulatory centers, particularly those mediated through the hypothalamus. These findings represent a promising step towards addressing adherence changes in patients with CPAP intolerance. Non-invasive, inexpensive, easily applicable, and cost-effective aromatic stimulation could be beneficial for these patients.

## Data Availability

The datasets used and/or analyzed during the current study are available from the corresponding author on reasonable request.

## Abbreviations

CPAP: Continuous Positive Airway Pressure
OSA: Obstructive Sleep Apnea
PSQI: Pittsburgh Sleep Quality Index
ESS: Epworth Sleepiness Scale
AHI: Apnea Hypopnea Index
UPPP: Uvulopalatopharyngoplasty
HGNS: Hypoglossal Nerve Stimulation

## References

[1] He J, Kryger MH, Zorick FJ, Conway X, Roth T. Mortality and apnea index in obstructive sleep apnea. Chest. 1988;94:9–14. 3289839

[2] Newman AB, Javier Nieto F, Guidry U, Lind BK, Redline S, T G Pickering TG, et al. Relation of Sleep-disordered Breathing to Cardiovascular Disease Risk Factors. Am J Epidemiol. 2001;154:1–12. 10.1093/aje/154.1.50 11434366

[3] Kasai T, Bradley TD. Obstructive sleep apnea and heart failure: pathophysiologic and therapeutic implications. J Am Coll Cardiol. 2011;57:119–27. 21211682 10.1016/j.jacc.2010.08.627

[4] Marin JM, Carrizo SJ, Vicente E, Agusti AGN. Long-term cardiovascular outcomes in men with obstructive sleep apnoea—hypopnoea with or without treatment with continuous positive airway pressure: An observational study. ACC Curr J Rev. 2005;14:8–9. 10.1016/S0140-6736(05)71141-7 15781100

[5] Floras JS. Obstructive sleep apnea syndrome, continuous positive airway pressure and treatment of hypertension. Eur J Pharmacol. 2015;763:28–37. 26096557 10.1016/j.ejphar.2015.06.024

[6] Patil SP, Ayappa IA, Caples SM, Kimoff RJ, Patel SR, Harrod CG. Treatment of adult obstructive sleep apnea with positive airway pressure: an American Academy of Sleep Medicine clinical practice guideline. J Clin Sleep Med. 2019;15:335–43. 30736887 10.5664/jcsm.7640 PMC6374094

[7] Campos-Rodriguez F, Peña-Griñan N, Reyes-Nuñez N, Cruz-Moron ID, Perez-Ronchel J, Vega-Gallardo FD, et al. Mortality in obstructive sleep apnea-hypopnea patients treated with positive airway pressure. Chest. 2005;128:624–33. 16100147 10.1378/chest.128.2.624

[8] Somiah M, Taxin Z, Keating J, Mooney AM, Norman RG, Rapoport DM, et al. Sleep quality, short-term and long-term CPAP adherence. J Clin Sleep Med. 2012;8:489–500. 23066359 10.5664/jcsm.2138 PMC3459193

[9] Wozniak DR, Lasserson TJ, Smith I. Educational, supportive and behavioural interventions to improve usage of continuous positive airway pressure machines in adults with obstructive sleep apnoea. Cochrane Database Syst. 2014;1:CD007736. 10.1002/14651858.CD007736.pub2. 10.1002/14651858.CD007736.pub2 24399660

[10] Ryan S, Doherty LS, Nolan GM, McNicholas WT. Effects of heated humidification and topical steroids on compliance, nasal symptoms, and quality of life in patients with obstructive sleep apnea syndrome using nasal continuous positive airway pressure. J Clin Sleep Med. 2009;05:422–7. PMC2762712 19961025

[11] Verbraecken J, Dieltjens M, Beeck SO, Vroegop A, Braem M, Vanderveken O, et al. Non-CPAP therapy for obstructive sleep apnoea. Breathe (Sheff). 2022;18:220164. 36340820 10.1183/20734735.0164-2022 PMC9584565

[12] Strollo PJ Jr, Soose RJ, Maurer JT, Vries N, Cornelius J, Froymovich O, et al. Upper-airway stimulation for obstructive sleep apnea. N Engl J Med. 2014;370:139–49. 24401051 10.1056/NEJMoa1308659

[13] Caples SM, Rowley JA, Prinsell JR, Pallanch JF, Elamin MB, Katz SG, et al. Surgical modifications of the upper airway for obstructive sleep apnea in adults: a systematic review and meta-analysis. Sleep. 2010;33:1396–407. 21061863 10.1093/sleep/33.10.1396 PMC2941427

[14] Okabe S, Fukuda K, Mochizuki-Kawai H, Yamada K. Favorite odor induces negative dream emotion during rapid eye movement sleep. Sleep Med. 2018;47:72–6. 29778917 10.1016/j.sleep.2018.03.026

[15] Knötzele J, Riemann D, Frase L, Bernd Feige, Elst LT, Kornmeier J. Presenting rose odor during learning, sleep and retrieval helps to improve memory consolidation: a real-life study. Sci Rep. 2023;13:2371. 10.1038/s41598-023-28676-z PMC9911722 36759589

[16] Karadag E, Samancioglu S, Ozden D, Bakir E. Effects of aromatherapy on sleep quality and anxiety of patients. Nurs Crit Care. 2017;22:105–12. 26211735 10.1111/nicc.12198

[17] McDonnell B, Newcomb P. Trial of essential oils to improve sleep for patients in cardiac rehabilitation. J Altern Complement Med. 2019;25:1193–9. 31556690 10.1089/acm.2019.0222

[18] Lewith GT, Godfrey AD, Prescott P. A single-blinded, randomized pilot study evaluating the aroma of *Lavandula augustifolia* as a treatment for mild insomnia. J Altern Complement Med. 2005;11:631–7. 16131287 10.1089/acm.2005.11.631

[19] Johns MW. A new method for measuring daytime sleepiness: the Epworth sleepiness scale. Sleep. 1991;14:540–5. 1798888 10.1093/sleep/14.6.540

[20] Rotenberg BW, Vicini C, Pang EB, Pang KP. Reconsidering first-line treatment for obstructive sleep apnea: a systematic review of the literature. J Otolaryngol Head Neck Surg. 2016;45:23. 27048606 10.1186/s40463-016-0136-4 PMC4822285

[21] Kuhn E, Schwarz EI, Bratton DJ, Rossi VA, Kohler M. Effects of CPAP and mandibular advancement devices on health-related quality of life in OSA: a systematic review and meta-analysis. Chest. 2017;151:786–94. 28130044 10.1016/j.chest.2017.01.020

[22] Anandam A, Patil M, Akinnusi M, Jaoude P, El-Solh AA. Cardiovascular mortality in obstructive sleep apnoea treated with continuous positive airway pressure or oral appliance: an observational study. Respirology. 2013;18:1184–90. 23731062 10.1111/resp.12140

[23] Hamoda MM, Kohzuka Y, Almeida FR. Oral appliances for the management of OSA. Chest. 2018;153:544–53. 28624182 10.1016/j.chest.2017.06.005

[24] Pang KP, Plaza G, Baptista JPM, Reina CO, Chan YH, Pang KA, et al. Palate surgery for obstructive sleep apnea: a 17-year meta-analysis. Eur Arch Otorhinolaryngol. 2018;275:1697–707. 29802464 10.1007/s00405-018-5015-3

[25] Kasai M, Minekawa A, Homma H, Nakazawa A, Iizuka T, Inoshita A, et al. Nasal surgery improves continuous positive airway pressure compliance and daytime sleepiness in obstructive sleep apnea syndrome. J Otol Rhinol. 2015; S1: 10.4172/2324-8785.s1-006.

[26] Kwak KH, Lee YJ, Lee JY, Cho JH, Choi JH. The effect of pharyngeal surgery on positive airway pressure therapy in obstructive sleep apnea: a meta-analysis. J Clin Med. 2022;11:6443. 36362672 10.3390/jcm11216443 PMC9658902

[27] Akashiba T, Inoue Y, Uchimura N, Ohi M, Kasai T, Kawana F, et al. Sleep apnea syndrome (SAS) clinical practice guidelines 2020. Sleep Biol Rhythms. 2022;20:5–37. 38469064 10.1007/s41105-021-00353-6 PMC10900032

[28] Heiser C, Steffen A, Boon M, Hofauer B, Doghramji K, Maurer JT, et al. Post-approval upper airway stimulation predictors of treatment effectiveness in the ADHERE registry. Eur Respir J. 2019;53:1801405. 30487205 10.1183/13993003.01405-2018 PMC6319796

[29] Gottfried JA. Central mechanisms of odour object perception. Nat Rev Neurosci. 2010;11:628–41. 20700142 10.1038/nrn2883 PMC3722866

[30] Arzi A, Shedlesky L, Ben-Shaul M, et al. Humans can learn new information during sleep. Nat Neurosci. 2012;15:1460–5. 22922782 10.1038/nn.3193

[31] Walliczek-Dworschak U, Cassel W, Mittendorf L, Pellegrino R, Koehler U, Güldner C, et al. Continuous positive air pressure improves orthonasal olfactory function of patients with obstructive sleep apnea. Sleep Med. 2017;34:24–9. 28522094 10.1016/j.sleep.2017.02.018

[32] Kaya KS, Akpınar M, Turk B, Seyhun N, Cankaya M, Coskun BS. Olfactory function in patients with obstructive sleep apnea using positive airway pressure. Ear Nose Throat J. 2020;99(4):239–44. 31565995 10.1177/0145561319878949

[33] Perl O, Kemer L, Green A, Arish N, Corcos Y, Arzi A, et al. Respiration-triggered olfactory stimulation reduces obstructive sleep apnea severity: a prospective pilot study. J Sleep Res. 2024;33:e14236. 38740050 10.1111/jsr.14236 PMC11597002

[34] Magliulo G, De Vincentiis M, Iannella G, Ciofalo A, Pasquariello B, Manno A, et al. Olfactory evaluation in obstructive sleep apnoea patients. Acta Otorhinolaryngol Ital. 2018;38:338–45. 30197425 10.14639/0392-100X-1981 PMC6146584

[35] Iannella G, Magliulo G, Maniaci A, Meccariello G, Cocuzza S, Cammaroto G, et al. Olfactory function in patients with obstructive sleep apnea: a meta-analysis study. Eur Arch Otorhinolaryngol. 2021;278:883–91. 32914257 10.1007/s00405-020-06316-w

[36] Japan Society of Otorhinolaryngology. Olfactory Dysfunction Clinical Practice Guideline, 2nd edition. Tokyo: Nankodo; 2025. [in Japanese] 10.7248/jjrhi.64.1

